# Anti-HBV Drugs: Progress, Unmet Needs, and New Hope

**DOI:** 10.3390/v7092854

**Published:** 2015-09-15

**Authors:** Lei Kang, Jiaqian Pan, Jiaofen Wu, Jiali Hu, Qian Sun, Jing Tang

**Affiliations:** 1 Department of Clinical Pharmacy, Shanghai First People’s Hospital, Shanghai Jiao Tong University, 650 New Songjiang Road, Songjiang District, Shanghai 201620, China; lakekang@163.com (L.K.); pan_jiaqian@163.com (J.P.); sq15895820107@126.com (Q.S.); 2 Department of Pharmacy, Ningbo Medical Treatment Center Lihuili Hospital, 57 Xingning Road, Ningbo 315040, China; xjtuwjf@163.com; 3 Department of Pharmacy, The Third Staff Hospital of Baogang Group, 15 Qingnian Road, Baotou 014010, China; hujiali1989@126.com

**Keywords:** hepatitis B virus, anti-HBV drugs, research and development, guidelines

## Abstract

Approximately 240 million people worldwide are chronically infected with hepatitis B virus (HBV), which represents a significant challenge to public health. The current goal in treating chronic HBV infection is to block progression of HBV-related liver injury and inflammation to end-stage liver diseases, including cirrhosis and hepatocellular carcinoma, because we are unable to eliminate chronic HBV infection. Available therapies for chronic HBV infection mainly include nucleos/tide analogues (NAs), non-NAs, and immunomodulatory agents. However, none of them is able to clear chronic HBV infection. Thus, a new generation of anti-HBV drugs is urgently needed. Progress has been made in the development and testing of new therapeutics against chronic HBV infection. This review aims to summarize the state of the art in new HBV drug research and development and to forecast research and development trends and directions in the near future.

## 1. Introduction

Hepatitis B virus (HBV) is a hepatotropic DNA virus that primarily infects hepatocytes and causes liver disease [1,2]. It is estimated that approximately 240 million people are chronically infected with HBV worldwide [3]. In a national survey in China, an HBV endemic country, the hepatitis B surface antigen (HBsAg) positive rate in the general population was reported to be about 10% [4]. Chronic HBV infection is a leading cause of chronic hepatitis and advanced-stage liver diseases, including cirrhosis and hepatocellular carcinoma (HCC). The pathogenesis of HBV infection remains poorly understood, and current HBV treatments are unsatisfactory. New efforts are being directed to develop new and more effective anti-HBV therapeutics [5,6,7,8,9]. This review reports the state of the art in research and development of new drugs against HBV.

## 2. HBV

### 2.1. Morphology and Viral Proteins

HBV infection produces a virion (also called Dane particle) that is 42 nm in size and subviral structures consisting of 22-nm spherical and filamentous particles [10]. The virion is composed of an outer shell and inner core. The virion shell (envelope) consists of HBsAg and phospholipids that are drawn from infected cells. The core consists of a nucleocapsid that contains 240 copies of HBV core protein and a partially double-stranded HBV genome. The HBsAg subviral particle does not contain the viral genome and is not infectious [11]. The HBV genome is 3.2 kb long and consists of four partially-overlapping open reading frames (ORFs) that encode pre-S/S, pre-core/core, Pol, and X proteins. The pre-S/S ORF encodes 163- and 226-amino acid pre-S and S (major envelope protein) peptides, respectively [12]. The pre-core/core ORF encodes HBeAg and HBcAg as well as polymerase protein. The X ORF encodes a 154-amino acid polypeptide (HBx).

### 2.2. Life Cycle

The HBV genome is partially double-stranded DNA. The viral P protein contains a RNA reverse transcriptase (RT) domain, which is required to reverse transcribe pregenomic RNA (pgRNA) to viral minus strand DNA. The HBV lifecycle involves many steps and has yet to be fully elucidated. The HBV virion binds to hepatocytes through cellular receptors, one of which has been identified as sodium taurocholate co-transporting polypeptide (NTCP) [13]. The virion, once it enters the cell, is decoated, and the released capsid is delivered to the nucleus to release the viral genome. The partially double-stranded DNA is converted to covalently closed circular DNA (cccDNA) to serve as a transcription template. Four RNA transcripts including 3.5-, 2.4-, 2.1-, and 0.7-kb viral RNAs are synthesized and migrate into the cytoplasm for translation of viral proteins. The pgRNA interacts with P and core proteins to form a nucleocapsid where the viral minus stranded DNA is reverse transcribed from the pgRNA. Plus stranded DNA is synthesized using the minus stranded DNA as a template to become a mature nucleocapsid. The mature capsid can be assembled into virions for the production of infectious viral particles or transported back to the nucleus to replenish the cccDNA pool (intracellular pathway), which is required for establishing and maintaining HBV infection. HBsAg synthesis occurs in the rough endoplasmic reticulum and is then transported to the Golgi apparatus for the assembly of virion and subviral particles [14]. Gaggar *et al.* [15] schematically described the key steps of the HBV lifecycle.

## 3. Current State of Anti-HBV Drugs

### 3.1. Immunoregulators

Immuno-regulator drugs [16] have already been used in treating severe pneumonia, immunodeficiency, and chronic hepatitis B. Many hepatitis B carriers, who are currently free of clinical hepatitis manifestations, can experience flare-ups of liver injury later. Immunoregulatory drugs can improve the patients’ immune response, especially the specific immunity to HBV. Immunoregulators may help the immune cells to recognize and destroy HBV-infected cells, resulting in clearance of HBV in those destructed cells.

#### 3.1.1. Interferon (IFN)

IFN [17] is a secretory glycoprotein and functions as an antiviral, anti-proliferation, and immune regulatory cytokine. Production of IFN is triggered when the host cells responsd to various stimuli. IFN is categorized as α-(white blood cells), β-(fibroblasts), and γ-(lymphocytes) based on the producing cells. Currently, the major type of IFN used to treat chronic HBV infection is the IFN that binds to specific receptors on the cell surface to trigger a series of signal transduction events, resulting in the production of antiviral protein (AVP), which degrades viral mRNA and inhibits viral replication. It also simultaneously strengthens the activity of natural killer (NK) cells, macrophages, and T lymphocytes, demonstrating that IFN regulates the immune response in addition to the direct antiviral ability.

The half-life of IFN can be extended through chemical modification. At present, polyethylene glycol (PEG) is the most widely used agent to form multiple copies of IFN molecules that are more slowly degraded once injected. The pegIFN acquires a longer half-life up to 40 hours and remains effective in inhibiting HBV replication for 168 h. Therefore, pegIFN only needs to be injected once a week, which makes compliance with the treatment schedule more convenient and easier for patients.

However, IFN treatment is expensive, and the adverse effects of both short- and long-acting IFNs are severe, including fever, hair loss, and reduction of white blood cells. In addition, the antiviral efficacy in patients with normal or mildly-elevated alanine aminotransferase (ALT) is poor.

#### 3.1.2. Thymosin-α1

Thymosin-α1 is one of the immunoregulators, and its main functions are to promote differentiation of T cells to a mature stage and enhance the response to antigens and other excitants. The action of boosting the host immune system helps the host mount a defense against chronic HBV infection, and thymosin-α1 also exhibits a modest antiviral effect. It can be combined with IFN to treat chronic hepatitis B. The guidelines of the Asian Pacific Association for the Study of the Liver (APASL) [18] reviewed recent clinical studies of thymosin-α1 and indicated that fixed therapeutic duration and minimal side effects are major advantages. More well-designed large-scale studies to confirm its efficacy and combination therapy with pegIFN or nucleoside analogues are direction for future development.

#### 3.1.3. Cytokines

Cytokines are synthetized and secreted by a multiple types of immune cells (such as monocytes, macrophages, T cells, B cells, and NK cells) and non-immune cells (such as endothelial cells, epidermal cells, and fibroblasts) upon stimulation. Cytokines are characterized by a small molecular size and extensive biological functions [19], with some showing strong antiviral activity. For instance, interleukin (IL)-12 can induce TO cells into T1 cells, leading to IFN production.

### 3.2. Nucleos/Tide Analogues (NAs)

#### 3.2.1. Lamivudine

Lamivudine, a pyrimidine nucleoside drug, was the first NA approved for treating chronic HBV infection. Lamivudine is a reverse transcriptase (RT) inhibitor and inhibits HBV replication by inhibiting RT activity to lower the HBV DNA level. The drug is orally administered with fast absorption and high bioavailability. It can effectively decrease the serum HBV DNA level in treated patients.

Lamivudine needs a long-term course to maintain inhibition. HBV replication returns back quickly once lamivudine is withdrawn. Another challenge to administration of lamivudine is a high frequency of resistance mutations even after a short course treatment [20,21]. The common drug resistant mutations include tyrosine-methionine-aspartate-aspartate mutations (known as the YMDD mutant).

#### 3.2.2. Telbivudine

Telbivudine is a specific, selective and oral drug used for treating chronic hepatitis B. It shows unique advantages in inhibition of the synthesis of both strands of HBV DNA. In clinical practice, telbivudine is the only drug that can be safely used for treating pregnant female patients. The antiviral efficacy of telbivudine is stronger than that of lamivudine. The number of patients with undetectable HBV DNA after telbivudine therapy is significantly higher than that after treatment with lamivudine [22,23].

#### 3.2.3. Entecavir

Entecavir is a guanine nucleoside drug that offers potent selective inhibition of HBV polymerase [18]. It becomes an active triphosphate form after phosphorylation in the cells. ETV inhibits all three functions of viral polymerase: (1) initiation of HBV polymerase; (2) synthesis of minus strand DNA from the pregenomic RNA template (RT step); and (3) synthesis of HBV DNA chain. The inhibitive efficacy of Entecavir is 300 times greater than that of other NAs, such as lamivudine or/and adefovir, and the resistance mutation frequency is very low [3,18,24]. It shares certain cross tolerance with lamivudine. The ETV regime should be fulfilled before cessation; otherwise, rapid deterioration may occur.

#### 3.2.4. Adefovir

Adefovir [25] is a purine nucleoside prodrug. Phosphorylation of adefovir is required for inhibition, and it occurs to form the active metabolite adefovir diphosphate once it enters the cell. Adefovir diphosphate can substitute for normal substrates of dATP (adenosine). Once incorporated into the viral DNA chain, it stops the elongation of viral DNA synthesis. It is suggested that adefovir can also induce production of α-IFN, increase NK cell activity, and stimulate the hosts’ immune response. Adefovir shows stronger efficacy in inhibiting HBV replication, but a lower resistance rate as an add-on therapy for LAM resistance than switching to ADV only [26,27]. A larger dose (30 mg or higher per day) can cause renal toxicity, but the standard dose at 10 mg/d does not affect renal function seriously [18]. Close monitoring of renal function is required when adefovir is in use.

#### 3.2.5. Tenofovir

Tenofovir is an acyclic adenine nucleotide analogue that inhibits both HBV and HIV replication at the RT step [3]. Tenofovir prodrug is converted to tenofovir diphosphate via catalysis by a cellular kinase. Tenofovir is recommended because of its potent inhibition, high barrier to drug resistance, and the overall balance between benefits and risks.

A summary of the structures the anti-HBV drugs are shown in Table 1, and the information is cited as provided in the manufacturers’ instructions.

### 3.3. Traditional Chinese Medicine

#### 3.3.1. *Sedum sarmentosum* Granules

*Sedum sarmentosum* granules are derived from either the fresh or dried whole plant of *sarmentosum*, which belongs to the *Crassulaceae* family. Investigation of effective pharmacological components has shown that the liver protective function is mainly based on constituents of megastigmane glycosides [28]. Experiments using an animal liver injury model have shown that *Sedum sarmentosum* granules can reduce serum ALT and aspartate aminotransferase (AST) levels in animals with acute liver injury [29]. In clinical practice, it is mainly used for treating acute or chronic hepatitis, especially patients whose cereal third transaminase level is increased [30]. In addition, *Sedum sarmentosum* granules can up-regulate cellular immunity even in a state of inhibition, which is crucial for facilitating recovery from liver injury.

At present, the mechanism of action and toxicology of this traditional Chinese medicine remain unknown, and therefore, reasonable dosages can only be empirically determined based on patients’ conditions.

**Table 1:** Summary of the structures of the anti-HBV drugs.

	Structures	Active Form Structures	EC_50_	Therapeutic Target
Lamivudine (EPIVIR-HBV [31])	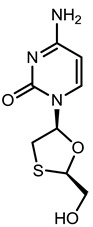	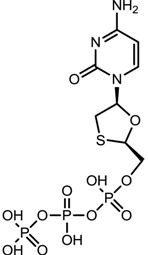	Varies from 0.01 μM (2.3 ng/mL) to 5.6 μM (1.3 μg/mL) depending upon the duration of exposure of cells to lamivudine, the cell model system, and the protocol used	Inhibition of the RNA- and DNA-dependent polymerase activities of HBV reverse transcriptase
Telbivudine (Sebivo [32])	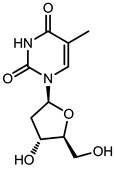	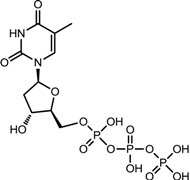	HBV first strand (EC_50_ = 0.4–1.3 μM) and second strand (EC_50_ = 0.12–0.24 μM)	Inhibition of HBV DNA polymerase (reverse transcriptase) by competing with the natural substrate, thymidine 5ʹ-triphosphate
Entecavir (Baraclude [33])	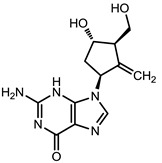	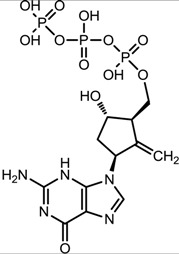	0.004 μM in human HepG2 cells transfected with wild-type HBV, 0.026 μM (range, 0.010–0.059 μM) against lamivudine resistance HBV (rtL180M and rtM204V)	Inhibition of (1) priming of the HBV polymerase; (2) reverse transcription of the negative strand DNA from the pregenomic messenger RNA; and (3) synthesis of the positive strand HBV DNA
Adefovir dipivoxil (SigmaPharm Laboratories, LLC [34])	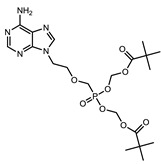	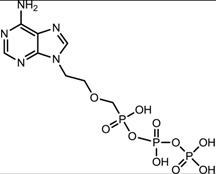	Ranges from 0.2 to 2.5 μM in HBV-transfected human hepatoma cell lines	Inhibition of HBV DNA polymerase (reverse transcriptase) by competing with the natural substrate deoxyadenosine triphosphate and by causing DNA chain termination after its incorporation into viral DNA
Tenofovir (VIREAD [35])	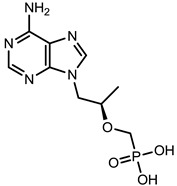	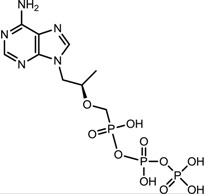	Ranges from 0.04 μM to 8.5 μM	Inhibition of the activity of HBV reverse transcriptase by competing with the natural substrate deoxyadenosine 5ʹ-triphosphate and, after incorporation into DNA, by DNA chain termination
Oxymatrine	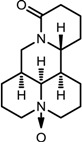	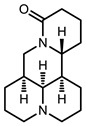	Unknown	Unknown

#### 3.3.2. Oxymatrine

Oxymatrine, an alkaloid extracted from the herb *Sophora alopecuraides* L, exhibits an anti-HBV effect in both HBV transgenic mice [36,37] and patients with chronic HBV infection [38], as shown in Table 1. Chen *et al.* [36] and Lu *et al.* [37] independently found that oxymatrine can suppress the levels of HBsAg and HBcAg in liver and HBV DNA in serum of transgenic mice. Recently, a clinical study [39] found that the combination of oxymatrine with lamivudine can prevent the development of lamivudine resistance in chronic HBV-infected patients. However, the antiviral mechanisms or targets of oxymatrine against HBV remain unknown. Xu *et al.* [40] suggested that oxymatrine may interfere with the packaging process of pgRNA into the nucleocapsid or suppress the activity of viral DNA polymerase. Wang *et al.* [41] found that oxymatrine may interfere with the reverse transcription process from pgRNA to DNA by destabilizing heat stress cognate 70 (Hsc70) mRNA.

Although oxymatrine was mentioned in the 2005 Chinese Guideline for Prevention and Treatment of Chronic HBV Infection [42], further longer-term, multicenter, and randomized, controlled clinical trials with large numbers of cases are needed to validate antiviral efficacy.

## 4. New Anti-HBV Drugs under Development and Evaluation

### 4.1. New Drugs that Target the Viral Components

#### 4.1.1. MCC-478

MCC-478, an adefovir derivative, has been in clinical Phase I trials for safety and efficacy assessment [43,44]. It acts similarly to other NAs and can inhibit HBV replication by inhibiting P protein packaging reaction. MCC-478 can be effective against both wild-type HBV and lamivudine-resistant mutants [45,46].

#### 4.1.2. cccDNA

Currently, the first-line antiviral therapy is NA-based treatment, which functions to suppress HBV replication. Although new cccDNA amplification is restrained, the hepatocytes remain infected due to persistent cccDNA in the nucleus, which escapes via the error-prone viral polymerase [47] and drug-resistance mutants [48]. Therefore, sustained elimination of cccDNA from infected hepatocytes represents a major challenge, and one possible solution is immunological therapy, such as cytokine-mediated or immune-associated receptor-mediated cccDNA degradation.

A very recent study by Lucifora *et al.* [49] demonstrated that high-dose IFN-α can induce cccDNA eradication in HBV-infected hepatocytes. Although the approved IFN-α therapy is effective in some patients, a relatively low response rate [50], contraindications, less convenient parenteral administration [51], and certain serious adverse effects [52] all limit its clinical application. Therefore, the better therapeutic option is the alternative receptor-mediated cccDNA degradation. Through the use of specific antibodies, lymphotoxin (LT) β receptor (LTβR) activation was also shown to induce cccDNA eradication in HBV-infected hepatocytes, without causing any detectable hepatocytotoxicity. With respect to the underlying mechanism, LTβR activation can up-regulate the expression of nuclear APOBEC3 (A3) deaminases and subsequently induce deamination and A purinic/A pyrimidinic (AP) site formation in HBV cccDNA, resulting in its degradation, without affecting host genomic DNA. The A3 family members of A3A and A3B, which are located in the nucleus [53], play an essential role in the eradication of foreign DNA [54,55]. They might be targeted to cccDNA by their interaction with the HBV core protein, suggesting a selective mechanism for distinguishing HBV cccDNA from host genomic DNA. Additionally, IFN-α was found to induce a similar effect, which indicates that, through use of LTβR agonists or adoptive T cell therapy [56], receptor-mediated cccDNA degradation, if confirmed in clinical trials, could lead to clearance of chronic HBV infection from the liver.

In addition, Ahmed *et al.* [57] summarized current therapeutic strategies against cccDNA production. In addition to INFs and LTβR agonists, factors (methylation and acetylation) affecting the process of cccDNA transcription and translation and DNA cleavage enzymes (zinc-finger protein nucleases and transcription-activator-like effectors), which interrupt the structure and/or functions of cccDNA, are some of the other possible approaches.

#### 4.1.3. HBsAg Gene

The major component of HBsAg is the small S protein with 226 amino acids. HBsAg elicits production of neutralizing antibodies. However, HBsAg is overwhelmingly produced and stably maintained in chronically HBV-infected patients, which contributes to the suppression of an HBV-specific immune response. In addition to the immune response, HBsAg is required for assembling viral particles. A group of researchers [58] investigated the cellular gene expression profile in cells containing transfected HBsAg gene via microarray analysis. It was found that among 1152 gene analyzed, 30 were significantly upregulated, including tumor necrosis factor (TNF)-related apoptosis-inducing ligand (TRAIL) receptor, cell division cycle protein (CDC23), and FKBP-associated protein (FAP48). Moreover, 29 genes were significantly down-regulated, including TNF receptor-associated factor (TRAF), TNF receptor-associated protein (AD022), and TNF receptor-associated factor 2 (TRAF2). These genes impact cell growth, apoptosis, signal transduction, immune regulation, and tumorigenesis. Both up- and down-regulation of specific proteins is involved in the process of apoptosis, suggesting that HBsAg is probably involved in the regulation of apoptosis. Furthermore, the HBsAg gene is thought to be involved in HCC development. Taken together, these findings suggest that HBsAg is a potential target for HCC gene therapy.

#### 4.1.4. Chinese Herbal Medicines

Helioxanthin (HE-145) is an arylnaphthalene ligand isolated from *Taiwania Cryptomerioides*. HE-145 and its analogues 5-4-2 [59], 8-1 [60], 32 [61], and 15 [62] have been reported to exhibit potent anti-HBV activity *in vitro*. They not only suppress the expression of HBV RNAs and proteins, but also suppress viral DNA replication of both wild-type and lamivudine-resistant mutants. The mode of action involves decreasing the DNA binding activity of hepatocyte nuclear extracts to specific *cis*-elements in the HBV core promoter, whereas the ectopic expression of the *cis*-elements relieves such suppression. Thus, HE-145 suppresses HBV gene expression and replication by selectively modulating the host transcriptional machinery [63].

HE-145 analogue 8-1 [60] reduces activity at all HBV promoters by post-transcriptionally decreasing the expression of critical transcription factors in HBV-producing cells, which diminishes their binding to the precore/core promoter enhancer II region. Thus, it blocks viral gene expression to negatively impact viral DNA replication.

Pang *et al.* [64] found that the ethanol extract from *Ampelopsis sinica* root (EASR) effectively suppresses the levels of HBsAg, HBeAg, and extracellular HBV DNA *in vitro* by selectively inhibiting the activities of several HBV promoters and the p53-associated signaling pathway.

### 4.2. New Drugs that Target Cellular Factors

#### 4.2.1. HBV Receptors

HBV is enveloped with viral envelope proteins. HBV envelope proteins not only protect the virus but also make it infectious as they are required for viral entry, a first step for initiating HBV infection. The viral entry is mediated by specific interactions between viral envelope proteins and receptors on hepatocytes. Studies have shown that the myristoylated preS1 domain of HBV L-protein plays a pivotal role in viral infectivity by mediating attachment to a hepatocyte-specific receptor [65,66]. Recently, one of the assumed HBV receptors was identified as sodium taurocholate cotransporting polypeptide (NTCP) [13]. NTCP-mediated HBV and HDV entry has been independently confirmed by other groups [67,68].

NTCP represents a new target for the development of therapeutics that can block HBV entry. Myrcludex-B, a synthetic lipopeptide derived from the HBV preS1 domain sequence, has been shown to specifically bind to NTCP. It can efficiently block *de novo* HBV infection and prevent intrahepatic viral spreading both *in vitro* and *in vivo* [67,69]. In addition, NTCP also functions as a hepatic bile acid transporter that mediates the uptake of most sodium-dependent bile salts into hepatocytes. Yan *et al.* [68] found that two uptake functions of NTCP seem to be mutually exclusive, implying that the regulation of uptake of bile acids or their derivatives could impact HBV entry, a potentially new strategy for the development of novel antiviral drugs.

NTCP expression is subjected to cellular regulation. It was suggested that retinoic acid receptor (RAR) regulates the promoter activity of the human NTCP (hNTCP) gene [70]. Tsukuda *et al.* [70] demonstrated that a RAR-selective antagonist Ro41-5253 decreases cellular susceptibility to HBV infection by inhibiting hNTCP promoter activity. Furthermore, IL-6 was found to regulate NTCP expression, and the effect of IL-6 on HBV entry was also noted. Bouezzedine *et al.* [71] found that NTCP mRNA expression is reduced by 98%, along with an 80% decrease in NTCP-mediated taurocholate uptake and 90% inhibition of HBV entry, upon pretreatment of HepaRG cells with IL-6. Such findings require further validation in more stringent infection systems.

Other cellular factors that are involved in the HBV lifecycle include the Toll-like receptors (TLRs). TLRs are known to mediate the innate immune response to infection. TLRs recognize pathogen-associated molecular patterns and respond by activating a series of antiviral mechanisms. The TLR-ligand interaction results in the production and release of antiviral molecules such as IFNs, pro-inflammatory cytokines, and chemokines. However, HBV can disrupt TLR expression and hinder intracellular signaling cascades as a strategy for evading the innate immune response to chronic HBV infection. An emerging new treatment strategy involves combining antiviral treatment with adjuvant therapy using a TLR agonist to restore the innate immune response. TLR ligands that can activate the TLR-mediated innate immune response may represent promising adjuvant drug candidates. Isogawa *et al.* [72] found that all ligands, except the one for TLR2, inhibit HBV replication in a IFN-α- and -β-dependent manner after ligands specific for TLR 2-5, 7, and 9 are individually administered to HBV transgenic mice, suggesting that ligand-TLR interaction can elicit an effective immune response to inhibit HBV replication.

GS-9620, an orally-administered agonist of TLR7, was investigated for its safety, tolerability, pharmacokinetics, and pharmacodynamics in healthy volunteers and hepatitis C virus (HCV)- and HBV-infected patients. Three phase I clinical trials were completed [73,74,75]. GS9620 was well-absorbed and tolerated at oral doses of 0.3, 1, 2, 4, 6, 8, or 12 mg per day. The tested dosages were finally adjusted to 0.3, 1, 2, and 4 mg because chemokines/cytokines and IFN-stimulated genes (ISGs) can be induced at doses ≥2 mg. The most common adverse events were flu-like symptoms and headache. In healthy volunteers, minimal adverse events were similar to symptoms associated with an increased serum IFN-α level. In the majority of HBV- and HCV-infected patients, adverse events varied from mild to moderate in severity and the serum IFN-α levels became detectable in 16.7% (8/48) and 12% of HCV and HBV patients, respectively. A transient dose-dependent ISG15 induction was observed, peaking within 48 hours and followed by a decrease to baseline within 7 days. However, there were no significant changes in HCV RNA in HCV-infected patients, and no significant reductions of the HBsAg or HBV DNA level in HBV-infected patients either.

Recently, Zhang *et al.* [76] described a subgroup of the TLR family and reported that TLR3 and TLR2/4-mediated innate immune responses can control HBV infection. Kapoor *et al.* [77] summarized the roles of TLR7 and TLR9 in chronic HBV infection.

#### 4.2.2. Novel Target: La Protein Inhibitor (HBSC11)

La protein [78] is a phosphoprotein with a molecular weight of 47 kDa. It was initially thought to be a self-antigen produced in patients with systemic lupus erythematosus (SLE) and primary Sjogren’s syndrome (pSS). Now, it is known that human La protein is a multifunctional RNA-binding protein that is also involved in HBV RNA metabolism. A previous study showed that La protein exhibits a protective effect against HBV, and the protein kinase CK2 (tyrosine kinase II) enables La protein to be phosphorylated at serine 366, which activates the La protein functions [79]. Recently, *in vitro* experiments have also shown that La protein is the HBV RNA transcription factor. It transfers the HBsAg-specific cytotoxic T cells (CLTs) to the liver of HBV-infected transgenic mice and degrades HBV RNA, which leads to the disappearance of mouse La protein [80]. Based on the findings described above La protein is involved in HBV replication. Researchers have utilized virtual screening techniques to filter the La protein binding sites through multi-level molecular docking and target screening process. HBSC11, a novel inhibitor that targets human La protein was shown to have an anti-HBV effect, via the use of the Specs database and laboratory chemical database. This *in vitro* validation shows that HBSC11 could potently inhibit the transcription and expression of La protein [81].

#### 4.2.3. Transforming Growth Factor-β (TGF-β)

The TGF-β superfamily consists of a group of bioactive polypeptides with related structures and similar functions in regulating cell growth, differentiation, migration, death, and extracellular matrix (ECM) production. Thus, TGF-β can regulate the growth and differentiation of endothelial cells, inflammatory cell chemotaxis, fibroblast proliferation, carcinogenesis, and ECM synthesis and degradation. Smad3, a key protein in the Smads signaling pathway, plays a positive role in the regulation of the TGF-β1 pathway, which is highly involved in organ fibrosis. In a clinical study [82], researchers detected an abnormally higher level of TGF-β1 in liver cancer tissue via immunohistochemistry. Furthermore, TGF-β levels were elevated in the liver cancer tissues, regardless of whether they represented primary or metastatic cancer. In addition, many studies have suggested that TGF-β1 is involved in the pathogenic process of hepatitis and liver fibrosis.

In an investigation of TGF-β1 expression in chronic HBV infection, Peng *et al.* [83] found that the serum TGF-β1 level increased gradually with the progressive severity of liver damage in 89 cases with mild, moderate, and severe chronic HBV infection. TGF-β1 can promote ECM synthesis and deposition, which is a pivotal factor for inducing liver fibrosis. Thus, it plays a key role in chronic HBV infection-induced liver inflammation and fibrosis. At present, many studies have also examined the molecular structure of TGF-β1 to define the relationship between TGF-β1 and HBV from a perspective of gene polymorphisms [84]. However, many of the results are inconsistent and unsatisfactory because of ethnic and geographical differences and case selection bias. The only consistent finding is that these gene polymorphism sites are mainly located in the promoter and control regions.

#### 4.2.4. MicroRNAs

MicroRNAs (miRNA) are small single-stranded RNAs with a final length of 20–23 bases. miRNAs are generally transcribed from non-coding regions of cellular genes. In 1993, a miRNA was first detected in *Caenorhabditis elegans*, and then in humans, plants, and other organisms. miRNAs are non-coding RNAs, but with regulatory functions at the mRNA and protein translational levels. miRNA regulation contributes to the control of physiological processes such as cell growth, differentiation, and apoptosis, lipid metabolism, and hormone secretion. In recent years, many studies have shown that a variety of human tumors are associated with aberrant expression of miRNAs. For instance, miRNA-221 is upregulated in pancreatic cancer. miRNA normally participates in the maintenance of cell homeostasis by regulating the target mRNA and its translation. Studies have indicated that miRNA expression is altered during cancer development. Abnormal expression of multiple miRNAs was detected in HCC cells. Furthermore, miRNA-199a-3p and miRNA-210 can effectively reduce the expression of HBsAg in HBV infection, indicating that miRNAs can not only regulate tumorigenesis but also mediate the interaction between the virus and the host [85]. However, no significant differences in miRNA expression were found between cirrhosis and HCC patients, suggesting that the abnormal expression of miRNAs already occurred in the early phase of the process. Changes in miRNA expression are presumed to be an initiating factor. However, the underlying mechanisms have yet to be clearly elucidated. There are two opinions: the most popular opinion is that miRNAs can degrade target mRNA molecules by complementary binding to the 3ʹ-end of the untranslated region (UTR) of the target mRNA. The other opinion is that miRNAs inhibit translation of the target mRNA to reduce the protein level of the targeted gene. Due to the fact that miRNAs are small molecules, lack immunogenicity, and exhibit diverse regulatory functions at the mRNA and protein translational levels, miRNAs directly degrade specific mRNAs. miRNAs can be used as a molecular tool to target HBV RNA to inhibit the HBV lifecycle. However, a challenge is that a single miRNA can have multiple targets, and this multi-specificity for target genes may limit the clinical application of a given miRNA.

### 4.3. Immune Checkpoints

Immune checkpoints refer to a homeostatic function of the immune system and are responsible for the balance of co-stimulatory and co-inhibitory signals [86]. Under normal physiological conditions, immune checkpoints play an essential role in maintaining self-tolerance, whereas upon pathogen infection, they function to regulate the amplitude and duration of immune responses [87]. Tumor cells and viruses can take advantage of these immune checkpoint pathways and exploit them for immune evasion. The two major immune checkpoint targets, cytotoxic T-lymphocyte antigen 4 (CTLA-4) and programmed cell-death protein 1 (PD1), both are negative immunomodulatory molecules and can inhibit T cell-mediated immune responses. Blocking of these two molecules is thought to prompt the immune system to regain strength to destroy the tumor cells. Ipilimumab (Yervoy; Bristol-Myers Squibb; Middlesex, UK), nivolumab (Opdivo; Bristol-Myers Squibb/Ono Pharmaceuticals; Middlesex, UK), and pembrolizumab (Keytruda; Merck & Co; Hertfordshire, UK.) are three immune checkpoint inhibitors currently approved for the treatment of malignant melanoma [86]. They are a CTLA-4- and PD1-specific monoclonal antibody and an anti-PD1 therapy, respectively. Expanding indications for other cancers and combination therapy [88] are the future directions for development of these drugs. In addition, a number of other immune checkpoint inhibitors are currently in the development pipeline [86].

However, the concept of blocking immune checkpoint inhibitors for HBV therapy is still in its infancy [89]. One important aspect is that blocking of immune checkpoint inhibitors should be a targeted therapy in order to avoid autoimmune-like side effects, as treatments targeting the T cell immunoglobulin-3 (TIM-3) pathway [90] and LTβR-mediated cccDNA degradation [49] do. Another important aspect is that the structural information and experimental data for the blocking agents should be understood as much as possible. The prospective from small molecules [91,92] and state-of-the-art technologies [93,94] can help to understand and expand the relevant knowledge. Therefore, based on oncological application (blocking agents) and the developing knowledge of immune checkpoint inhibitors, it is hoped that the application of certain blocking agents for treating chronic viral disease will not be too far in the future [89,95].

## 5. Guidelines for Currently Approved Medications

In 2015, the World Health Organization (WHO) published guidelines for the prevention of HBV infection and care and treatment of persons with chronic HBV infection [3]. The WHO guidelines recommend that the NAs tenofovir and entecavir, which have a high genetic barrier to drug resistance, should be the first-line treatments in all patients over 12 years of age, and entecavir should be used for children aged 2–11 years. Tenofovir is recommended as the second-line treatment in the same pediatric group. Other NAs (such as lamivudine, adefovir, and telbivudine) with a low barrier to drug resistance are explicitly not recommended. In addition, IFN is not considered a treatment option due to resource-limited settings and contraindications.

Before the WHO guidelines were published, the European Association for the Study of the Liver (EASL) [24] and the Asian Pacific Association for the Study of the Liver (APASL) [18] separately released their guidelines for the treatment of chronic HBV infection.

Lamivudine, entecavir, telbivudine, adefovir, tenofovir, and pegIFN have been approved in Europe for HBV treatment. The EASL guidelines of 2012 provided two treatment strategies, including finite-duration treatment with pegIFN and a long-term treatment with NAs. pegIFN, if chosen, should be used with caution due to the contraindications, and its combination with NAs is not recommended. The NA used for finite-duration treatment should be the most potent agent with the highest barrier to drug resistance. Tenofovir and entecavir are the first-line monotherapies. The evidence shows that the vast majority patients with monotherapy for ≥3 years maintain a full virological response [96,97]. Solutions to antiviral treatment failure are also recommended in the WHO guidelines. In case of primary non-response or partial response, a drug switch to tenofovir or entecavir from the initial drug is recommended. Once virological breakthrough is detected and incomplete patient compliance is excluded as a possible cause, a new therapeutic regimen should be adopted as early as possible after monitoring HBV DNA loads and identifying the pattern of resistance mutations.

The 2012 APASL guidelines state that HBV does not directly lead to cytopathic consequences and chronic HBV infection is a dynamic process of interaction among the virus, hepatocytes, and the host immune system. Therefore, chronic HBV infection therapeutics include immunomodulatory agents and antivirals used with NAs. The immunomodulatory agents include conventional IFN-α, pegIFN, and thymosin α1. The listed NAs are lamivudine, telbivudine, adefovir, tenofovir, and entecavir. Entecavir or tenofovir is the first-line drug, whereas the others are considered as second-line drugs. Notably, cost is still one of the most important factors for drug selection aside from the drug efficacy in the Asia-Pacific region. This restraint can be only addressed by developing more effective, but less expensive, new drugs.

## 6. Conclusions

In summary, the number of patients chronically infected with HBV continues to grow, and chronic HBV infection can lead to cirrhosis and HCC, causing an unbearable burden to patients and society. Current antiviral therapies can potently inhibit HBV replication and improve liver pathology but are rarely able to clear chronic HBV infection. Both new antiviral strategies and drugs are urgently needed. Several new and encouraging drug candidates are under development, but much research is still needed before they can be applied clinically.
